# Trends and Disparities in Peripheral Artery Disease and Dementia‐Related Mortality in the United States, 1999–2024

**DOI:** 10.1002/brb3.71912

**Published:** 2026-11-09

**Authors:** Naveed Ahmad, Syed Tawassul Hassan, Hajra Hameed, Muhammad Owais Shaikh, Kaneez Fatima, Aneeza Hameed, Muhammad Affaan Ansari, Ali Jan Yaqubi

**Affiliations:** ^1^ Khyber Medical College Peshawar Khyber Pakhtunkhwa Pakistan; ^2^ Karachi Medical and Dental College Karachi Metropolitan University Karachi Sindh Pakistan; ^3^ Islamic International Medical College (IIMC) Rawalpindi Punjab Pakistan; ^4^ Liaquat University of Medical and Health Sciences (LUMHS) Jamshoro Sindh Pakistan; ^5^ CMH Institute of Medical Science (CIMS) Multan Punjab Pakistan; ^6^ Wah Medical College Wah Cantonment Punjab Pakistan; ^7^ Dow University of Health Sciences (DUHS) Karachi Sindh Pakistan; ^8^ Kabul University of Medical Sciences Abu Ali Ibn Sina Kabul Afghanistan

**Keywords:** CDC WONDER, dementia, Joinpoint regression, mortality trends, peripheral artery disease

## Abstract

**Background:**

Cardiovascular risk factors are common among individuals with both peripheral artery disease (PAD) and dementia, and these co‐existing conditions are a major public health problem. Our goal is to assess nationwide mortality trends and disparities associated with concomitant PAD and dementia in the United States between 1999 and 2024.

**Methods:**

This is a population‐based analysis using the CDC WONDER Multiple Cause of Death database to assess adults aged 65 years and older. Death certificates that mentioned both PAD and dementia were included. Age‐adjusted mortality rates (AAMRs) and annual percent changes (APCs) were calculated to study the national trends by demographic and geographic subgroups.

**Results:**

There were 317,390 deaths among patients who had both PAD and dementia. The overall AAMR was fairly stable over the study period, rising marginally from 23.97 in 1999 to 24.22 in 2024. AAMRs were higher in non‐Hispanic African Americans across racial and ethnic groups and peaked in non‐metropolitan regions. The sensitivity analyses showed that mortality rates that were driven by PAD significantly declined (AAPC: −4.77) and rates driven by dementia (AAPC: 3.77) increased significantly over the 25‐year period.

**Conclusions:**

Overall, the age‐standardized mortality for concomitant PAD and dementia was largely stable, but there are still important geographical and demographic differences. The mortality curves when divided by underlying cause of death show that deaths due to PAD have decreased, while deaths due to dementia have risen in the United States among older adults over the study period.

## Graphical abstract

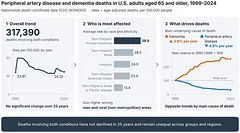

## 1 Introduction

Peripheral artery disease (PAD) is one of the major clinical presentations of systemic atherosclerosis, affects an estimated 202 million people worldwide, and is a significant contributor to cardiovascular morbidity and mortality in the United States (Fowkes et al. 2013; Criqui and Aboyans 2015). PAD is characterized by reduced blood flow to the arteries of the lower extremities, recurrent hospitalizations, poor quality of life, and an increased risk for cardiovascular conditions such as heart attack, stroke, or even premature mortality (Criqui and Aboyans 2015; Gerhard‐Herman et al. 2017). Although there have been evolving revascularization strategies and increased use of guideline‐directed medical therapies, PAD still carries an enormous burden for patients, healthcare systems, and society (Tsao et al. 2023). Meanwhile, dementia, including Alzheimer's disease and other neurodegenerative or vascular dementias, is recognized as one of the most pressing public health problems of the twenty‐first century. There are about 6.7 million people with Alzheimer's dementia in the United States who are aged 65 years and above. This is one of the leading causes of disability and mortality in the United States, and the cost to the healthcare system for patients with dementia is estimated to be $345 billion by 2023 (Alzheimer's Association 2023). Further, it is projected that the number of individuals with dementia will almost triple by 2050 across the globe, owing to the predicted increase in aged populations throughout the world (GBD 2019 Dementia Forecasting Collaborators 2022).

The association between PAD and dementia is biologically plausible and clinically significant as both of them share common cardiovascular risk factors such as advanced age, hypertension, diabetes mellitus, smoking cigarettes, dyslipidemia, obesity, and chronic low‐grade inflammation (Livingston et al. 2020). Atherosclerosis, endothelial dysfunction, and vascular injury, pathophysiological hallmarks associated with PAD, may cause dysfunction of cerebral autoregulation, white matter hyperintensities, and the activation of a neuroinflammatory pathway involved in vascular dementia (Iadecola et al. 2019; Dichgans and Leys 2017). Furthermore, reduced ankle–brachial index, which is the cornerstone for diagnosis of PAD, has been independently linked with elevated risks of cognitive decline and mortality in population‐based cohort studies (Iadecola et al. 2019; Collaboration et al. 2008). On the other hand, having dementia can influence the PAD prognosis adversely due to failure to recognize symptoms, noncompliance with medical and behavioral treatment regimens, progressive physical fragility, and late referral for care (Livingston et al. 2020; Iadecola et al. 2019). The coexistence of these two diseases may potentially create a synergistic adverse impact on health outcomes, which will contribute more to the creation of disabilities, hospitalizations, and mortality.

Existing evidence has established a robust relationship between PAD and poor cardiovascular health outcomes, and also between cerebrovascular disease and rapid cognitive decline (Criqui and Aboyans 2015; Dichgans and Leys 2017). However, the majority of previous investigations have relied on hospital registry data, administrative databases, or geographically restricted clinical samples that limit the generalization of the findings to the entire nation. Currently, we lack national‐level population data that analyze long‐term mortality patterns associated with the combined presence of PAD and dementia. In addition, no study has explored the dementia‐ and PAD‐related mortality disparities across nationwide demographic and geographic subgroups spanning decades. Hence, a comprehensive nationwide mortality analysis utilizing death certificates is essential in understanding the contemporary burden of this condition in modern times as well as identifying high‐risk populations that should be the focus of preventive strategies and treatment. Therefore, we aimed to assess mortality trends associated with PAD and dementia in the United States from 1999 to 2024 using the Centers for Disease Control and Prevention Wide‐ranging Online Data for Epidemiologic Research (CDC WONDER) Multiple Cause of Death database. The data were stratified to include demographics like sex and ethnicity, and geography like region of residence, degree of urbanization, and the specific US state.

## 2 Methods

### 2.1 Study Design and Data Sources

We conducted a population‐based, retrospective study using mortality registries from 1999 to 2024 to examine the mortality burden associated with concurrent PAD and dementia. The datasets were extracted from the CDC WONDER Multiple Cause‐of‐Death database and included decedents aged ≥ 65 years (National Center for Health Statistics 2026). Our investigation was fully based on anonymized mortality data available in the public domain, and no direct human subjects were involved, so approval from the institutional review board and the informed consent of the patients were not required (Human Research Protection Program 2026). The study design adhered to STROBE reporting guidelines and was based on the ethical standards of the Declaration of Helsinki (von Elm et al. 2007; World Medical Association 2013).

### 2.2 Case Identification and Variable Stratification

Case identification was performed using the 10th Revision of the International Statistical Classification of Diseases and Related Health Problems (ICD‐10) and an operational definition was employed for a broad category of peripheral and arterial diseases using the diagnostic codes for PAD and related vascular conditions (E10.5, E11.5, E12.5, E13.5, E14.5, I70.0–I70.2, I70.8–I70.9, I71.1–I71.6, I71.8–I71.9, I72.1–I72.4, I72.8–I72.9, I73.8–I73.9, I74.0, I74.1–I74.5, I74.8–I74.9, and I77.8–I77.9) and dementia (F01, F03, G30, and G31) (World Health Organization 2016; Issa et al. 2023; Khan et al. 2025). This expanded coding strategy, which includes diabetic peripheral circulatory complications, aneurysms, and arterial embolism/thrombosis, was purposefully chosen because the atherosclerotic burden is often systemic, and this strategy better captures the generalized vascular disease commonly seen in neurodegenerative diseases among older adults. However, the death certificates where both conditions were mentioned as either underlying cause of death (UCD) or as multiple/contributing cause of death (MCD) were selected for the final cohort inclusion. Furthermore, the study population was limited to adults aged ≥ 65 years, as this demographic is most heavily affected in regard to prevalence and mortality for both PAD and dementia. Rates reported as “suppressed” (< 10 deaths) or “unreliable” (< 20 deaths) by the CDC were treated following the standard NCI recommendations and were not included in statistical significance testing to prevent data instability. The demographic and geographic factors were classified systematically across several dimensions. Three age groups were sampled to describe age profiles (65–74, 75–84, and 85 years or older), and racial/ethnic groups included non‐Hispanic (NH) White, NH African American, Hispanic, NH American Indian, and NH Asian or Pacific Islander. Geographic data were mapped over all 50 states, including the District of Columbia, and these data sets were further aggregated to the four major geographic regions of the US Census Bureau: Northeast, Midwest, South, and West (United States Census Bureau 2021). To examine the differences between urbanization levels, metropolitan (large central, large fringe, medium, and small), and nonmetropolitan (micropolitan and noncore) designations were used in the analysis, based on the 2013 scheme of the National Center for Health Statistics (Ingram and Franco 2014). Also, we collected information that identified the patient's place of death (inpatient or outpatient medical facility, hospice, nursing homes, long‐term care homes, or private home).

### 2.3 Statistical Analysis

The description of the mortality trends was quantified using crude mortality rates (CMRs) and age‐adjusted mortality rates (AAMRs) per 100,000 population, and the 2000 US standard population was taken as a baseline during rate computation (Anderson and Rosenberg 1998). The annual percent change (APC) and average annual percent change (AAPC) that are used to assess temporal shifts in mortality were modeled using Joinpoint regression software (V 5.4.0.0, National Cancer Institute) alongside their respective 95% confidence intervals (CIs) (National Cancer Institute 2025). For the Joinpoint regression models, we permitted a minimum of 0 and a maximum of 4 Joinpoints, utilizing the Grid Search method. For the primary analyses, model selection was performed using the permutation test (overall significance level 0.05; 4,499 permutations), and 95% CIs for APC and AAPC estimates were calculated using the parametric method. For the sensitivity analyses, model selection was performed using the data‐driven weighted Bayesian information criterion (Weighted BIC), and 95% CIs were generated using the empirical quantile method with 5,001 resamples. The assumptions underlying the estimation of APC were met, assuming a constant rate of change within each linear segment. No formal adjustment for multiple testing (e.g., Bonferroni correction) was performed due to the exploratory and descriptive nature of this nationwide trend analysis; however, to maintain transparency, we reported the p‐values; nevertheless, the findings should be interpreted as exploratory. Two‐tailed *t*‐tests were used to assess whether these trend deviations were statistically significant at a predetermined alpha level of 0.05, corresponding to a slope significantly different from zero. Lastly, a sensitivity analysis was performed to confirm our findings with four different subgroups of isolated UCD‐PAD, isolated UCD‐dementia, UCD‐PAD + MCD‐dementia, and UCD‐dementia + MCD‐PAD.

## 3 Results

### 3.1 Proportional Mortality Rates

A total of 317,390 deaths occurred among patients with PAD and dementia. Females accounted for a higher proportion of deaths than males (62.70% vs. 37.30%). Regionally, the South contributed the largest share of deaths (35.52%), followed by the Midwest (25.42%), the West (22.99%), and the Northeast (16.07%). Among racial/ethnic groups, NH Whites accounted for the highest proportion of deaths (82.15%), followed by NH African Americans (10.46%), Hispanic individuals (5.38%), NH Asian or Pacific Islanders (1.66%), and NH American Indians (0.34%), who contributed the fewest deaths. Most deaths occurred in nursing homes/long‐term care facilities (56.6%), followed by the decedent's home (18.0%), medical facility inpatient settings (13.5%), other locations (5.5%), hospice facilities (3.6%), and medical facility outpatient/ER settings (2.4%). Less than 1% of deaths occurred among those dead‐on arrivals (0.2%), at unknown locations (0.2%), or in medical facilities with unknown status (0.1%) (Figure S1) (Table S1).

### 3.2 Year and Sex

There was no significant net long‐term change in the overall mortality rate across the complete study period, though distinct temporal fluctuations occurred. The AAMR was 23.97 in 1999 and 24.22 in 2024 (AAPC: −0.23; *p = *0.786). Mortality rates showed a notable decline from 2001 to 2015 (APC: −4.07*; *p* < 0.001). By sex, males consistently exhibited slightly higher AAMRs in recent years, rising from 22.94 in 1999 to 24.63 in 2024, while females showed a relatively stable pattern from 24.07 to 23.66 over the same period. Among males, a significant decline was observed between 2001 and 2012 (APC: −4.26*; *p* < 0.001). Similarly, females demonstrated a significant decrease from 2001 to 2015 (APC: −4.41*; *p* < 0.001). Overall, no statistically significant long‐term trend was observed in either sex (AAPC male: −0.07; *p = *0.937 vs. female: −0.34; *p = *0.684) (Figure 1) (Tables S2–S3).

**FIGURE 1:**
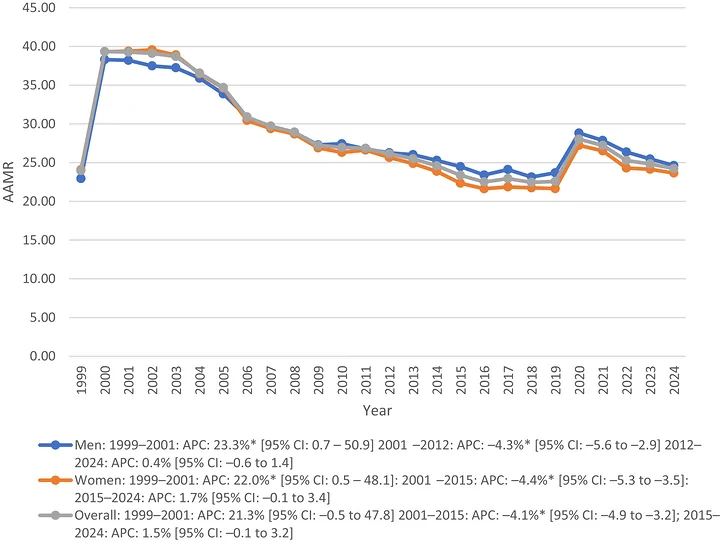
Overall and sex‐stratified peripheral artery disease and dementia‐related age‐adjusted mortality rates per 100,000 among older adults in the United States, from 1999 to 2024. Abbreviations: AAMR = age‐adjusted mortality rate; AAPC = average annual percent change; APC = annual percent change; CI = confidence interval. *Indicates that the annual percent change (APC) is significantly different from zero at α=0.05.

### 3.3 Race

The average mortality rate was highest among NH African Americans (38.9), followed by NH Whites (28.5), Hispanic individuals (24.0), NH American Indians (23.4), and NH Asian and Pacific individuals (13.4). Among NH American Indian individuals, mortality significantly decreased from 2000–2017 (APC: −3.74*; *p* < 0.001), and a significant decline from 2020 to 2024 (APC: −9.51*; *p = *0.019). NH Asian or Pacific Islander individuals showed a significant decline from 2001 to 2017 (APC: −3.01*; *p* < 0.001), followed by a notable increase from 2017 to 2022 (APC: 7.63*; *p = *0.010). NH African American individuals experienced a notable decline from 2002 to 2015 (APC: −5.07*; *p* < 0.001). NH White individuals showed a significant decline from 2001 to 2014 (APC: −4.27*; *p* < 0.001), and a subsequent increase from 2014 to 2024 (APC: 1.40*; *p = *0.039). Hispanic individuals demonstrated a sustained decline from 1999 to 2018 (Figure 2) (Tables S3–S4).

**FIGURE 2:**
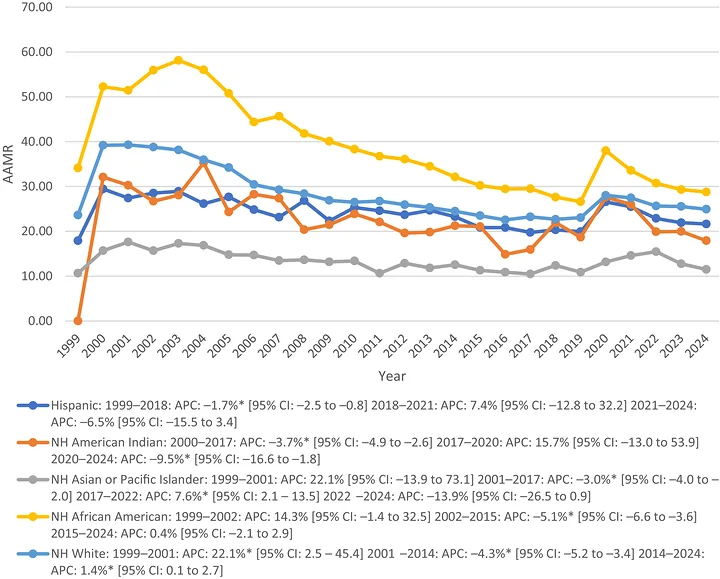
Peripheral artery disease and dementia‐related age‐adjusted mortality rates per 100,000, stratified by race/ethnicity among older adults in the United States, from 1999 to 2024. Abbreviations: AAMR = age‐adjusted mortality rate; AAPC = average annual percent change; APC = annual percent change; CI = confidence interval. *Indicates that the annual percent change (APC) is significantly different from zero at α=0.05.

### 3.4 Census Region

The AAMR varied across the US Census regions, with the Midwest (31.56) and West (30.83) showing higher overall rates compared with the South (28.89) and Northeast (22.02). In the Northeast, a significant decline in AAMR was observed from 2001 to 2007 (APC: −5.30*; *p = *0.026), followed by a continued but slower significant decline from 2007 to 2024 (APC: −1.24*; *p = *0.003). In the Midwest, AAMR increased significantly in 1999–2001 (APC: 29.17*; *p = *0.002), followed by a pronounced decline from 2001 to 2012 (APC: −5.43*; *p* < 0.001). In the South, mortality rates declined significantly from 2001 to 2015 (APC: −4.67*; *p* < 0.001), followed by a significant increase from 2015 to 2024 (APC: 2.47*; *p = *0.006). In the West, AAMR showed multiple fluctuations, including a significant rise from 1999 to 2001 (APC: 32.12*; *p* < 0.001), a decline from 2001 to 2007 (APC: −6.60*; *p* < 0.001). It continued to decrease from 2007 to 2018 (APC: −1.17*; *p = *0.003), followed by a significant increase from 2018 to 2021 (APC: 8.98*; *p = *0.037) and a subsequent decline from 2021 to 2024 (APC: −6.28*; *p = *0.004) (Figure 3) (Tables S3 and S5).

**FIGURE 3:**
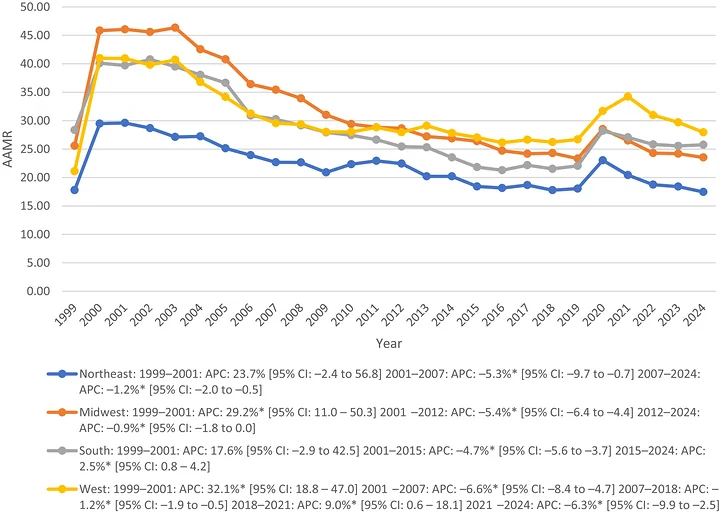
Peripheral artery disease and dementia‐related age‐adjusted mortality rates per 100,000, stratified by census regions among older adults in the United States, from 1999 to 2024. Abbreviations: AAMR = age‐adjusted mortality rate; AAPC = average annual percent change; APC = annual percent change; CI = confidence interval. *Indicates that the annual percent change (APC) is significantly different from zero at α=0.05.

### 3.5 Urbanization

Comparatively, non‐metropolitan areas had a higher average AAMR than metropolitan areas (32.37 vs. 28.38). Mortality rates increased significantly during 1999–2001 in metropolitan areas (APC: 26.22*; *p* < 0.001), followed by declines from 2001 to 2018, before rising again during 2018–2020 (APC: 11.92*; *p = *0.009). In non‐metropolitan areas, rates remained stable from 1999 to 2001 and then declined significantly from 2001 to 2020 (APC: −2.75*; *p* < 0.001) (Figure 4) (Tables S3 and S6).

**FIGURE 4:**
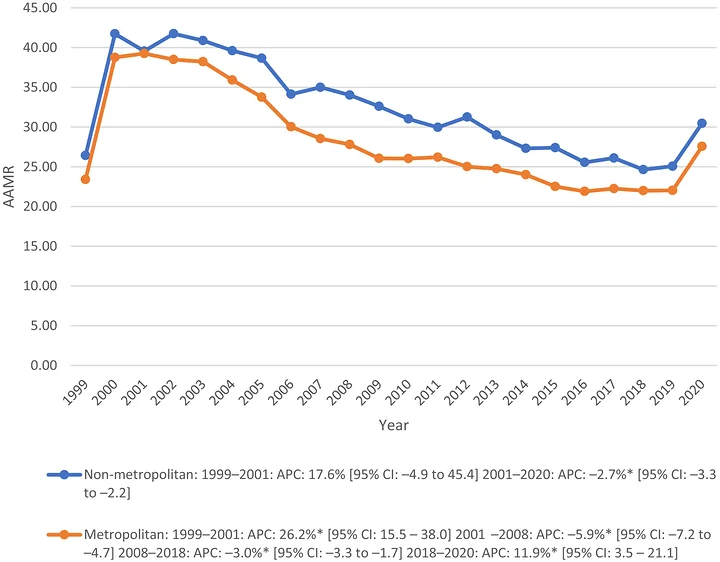
Peripheral artery disease and dementia‐related age‐adjusted mortality rates per 100,000, stratified by urbanization among older adults in the United States, from 1999 to 2020. Abbreviations: AAMR = age‐adjusted mortality rate; AAPC = average annual percent change; APC = annual percent change; CI = confidence interval. *Indicates that the annual percent change (APC) is significantly different from zero at α=0.05.

### 3.6 State

Ohio had the highest AAMR (48.50) in the year 2020, while Nevada had the lowest AAMR (14.31). The 10 states with the highest AAMR rankings were Ohio, Oklahoma, Tennessee, Vermont, Texas, West Virginia, Maryland, Rhode Island, Washington, and Minnesota, showing the greatest mortality burden. Conversely, the ten lowest ranking states (Nevada, Louisiana, Arkansas, Massachusetts, Florida, New York, Hawaii, Wyoming, Arizona, and New Jersey) had relatively low mortality rates (Figure 5) (Table S7). Geographic extremes should be interpreted in light of the fact that some states with relatively small numbers of total deaths may have inherently unstable AAMRs.

**FIGURE 5:**
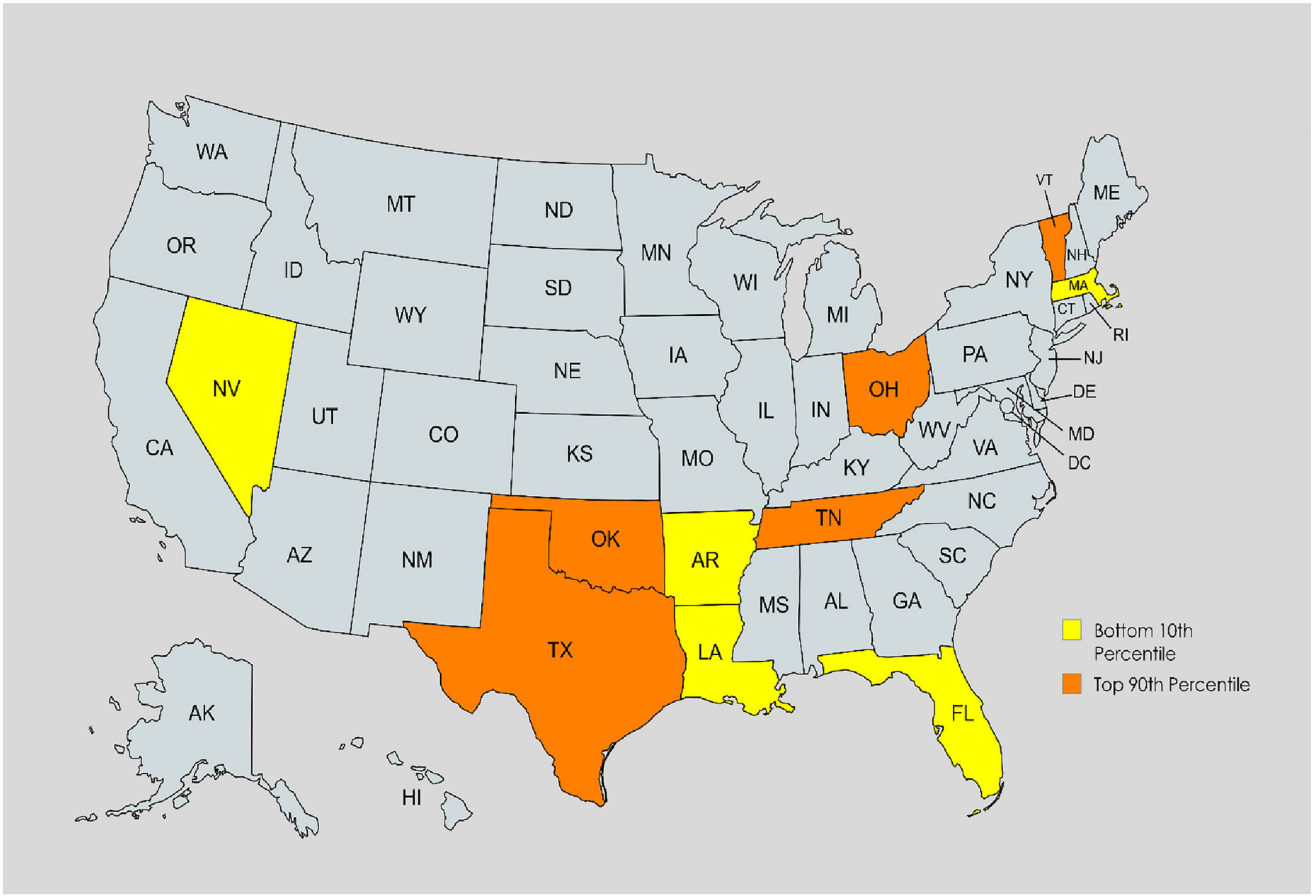
Peripheral artery disease and dementia‐related age‐adjusted mortality rates per 100,000, stratified by States among older adults in the United States, 2020. Abbreviations: AAMR = age‐adjusted mortality rate.

### 3.7 Sensitivity Analysis: Underlying Versus Contributing Causes of Death

A sensitivity analysis was conducted to test the consistency of primary findings by isolating mortality trends based on UCD and MCD classifications.

### 3.8 PAD‐Driven Mortality

The AAMR decreased significantly from 114.54 in 1999 to 34.63 in 2024 (AAPC: −4.77*, *p* < 0.001) among individuals listing PAD as UCD. This decline was accentuated by a large reduction from 2004 to 2011 (APC: −8.63*, *p* < 0.001), followed by a more gradual decline through 2024. However, individuals listing PAD as the UCD and dementia as an MCD also showed similar trends, with AAMRs decreasing greatly from 7.90 to 2.86 during the study period (AAPC: −4.61*, *p* < 0.001). Temporal trend analysis showed a sharp decrease from 2004 to 2007 (APC: −18.95*, *p* < 0.001) before settling into a steady downward trajectory (Figure 6A,B) (Tables S3 and S8).

**FIGURE 6:**
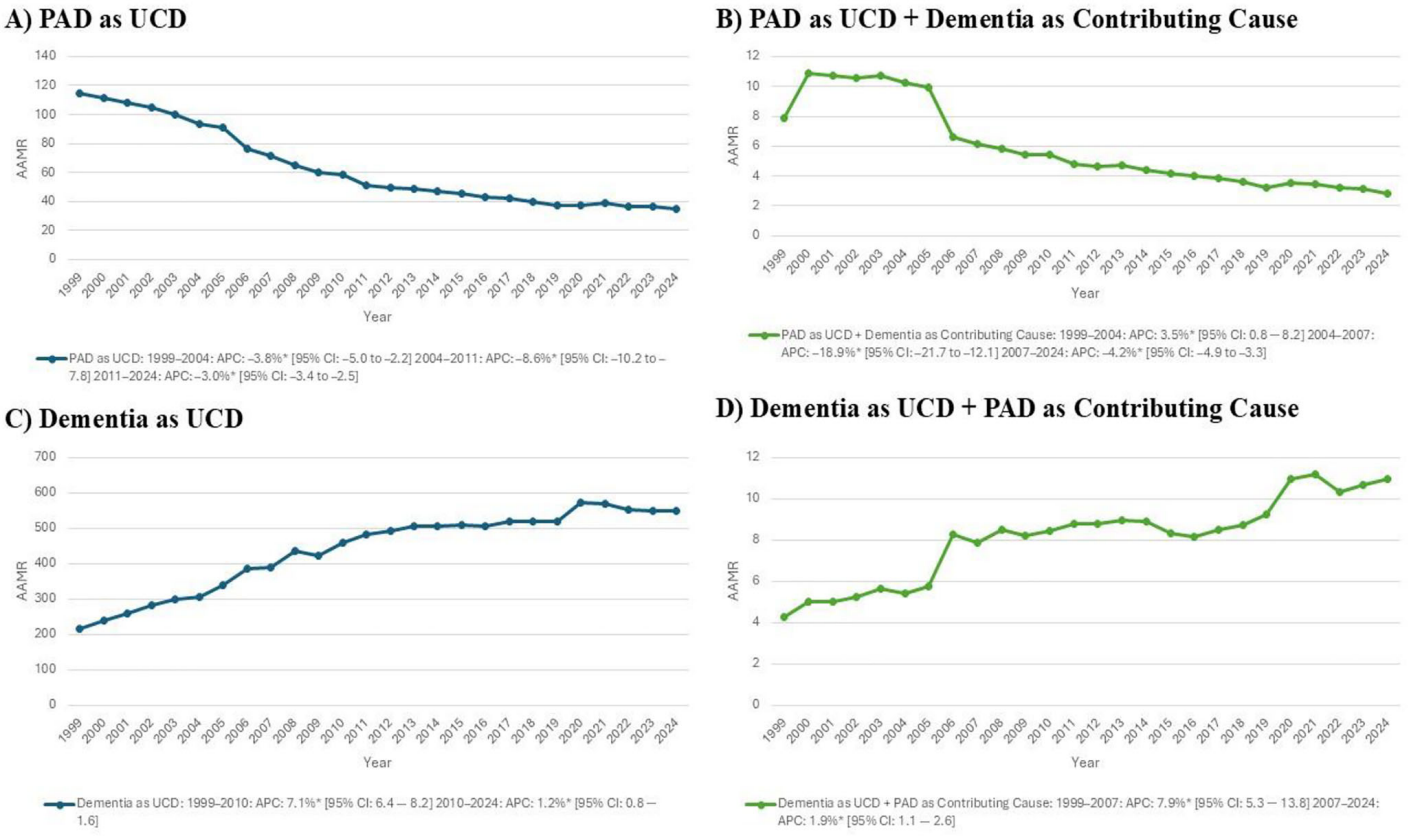
Sensitivity analysis of peripheral artery disease and dementia‐related age‐adjusted mortality rates per 100,000 among older adults in the United States, from 1999 to 2024, stratified by underlying and contributing causes of death. Abbreviations: AAMR = age‐adjusted mortality rate; AAPC = average annual percent change; APC = annual percent change; CI = confidence interval; MCD = multiple/contributing cause of death; UCD = underlying cause of death. *Indicates that the annual percent change (APC) is significantly different from zero at α=0.05.

### 3.9 Dementia‐Driven Mortality

Conversely, mortality rates driven primarily by dementia surged over the 25‐year period. The AAMR increased more than twofold from 214.21 in 1999 to 548.87 in 2024 (AAPC: 3.77*, *p* < 0.001) when dementia was classified as the UCD. This growth was the most rapid prior to 2010 (APC: 7.15*, *p* < 0.001), after which it continued to climb at a slower pace. Similarly, deaths attributed to UCD‐Dementia with contributing PAD increased from 4.26 to 10.96 (AAPC: 3.80*, *p* < 0.001). This cohort experienced significant growth from 1999 to 2007 (APC: 7.89*, *p* < 0.001), followed by a continued but slower significant increase from 2007 to 2024 (APC: 1.94*, *p = *0.006) (Figure 6C,D) (Tables S3 and S8).

## 4 Discussion

Using the CDC WONDER database, our study found a considerable mortality burden associated with PAD and dementia among older adults in the United States, characterized by 317,390 deaths, which disproportionately affected females, people residing in nursing homes, and populations from the South. Although such a significant burden of mortality was observed, no marked changes were noticed in terms of AAMR, which decreased significantly, followed by stabilization in recent years. This trend could also suggest enhanced management of cardiovascular risk factors in the treatment of PAD and dementia. However, this remains inconclusive in this current mortality‐focused analysis. It makes it biologically plausible for both PAD and dementia to occur together, considering the existence of common comorbidities and a shared pathophysiologic basis, such as atherosclerosis, endothelial dysfunction, inflammatory processes, and cerebral/peripheral hypoperfusion. Furthermore, dysfunctional vessels could affect the small brain vessels, causing injury, disruption of the blood‐brain barrier function, and cognitive impairment. These provide a possible mechanism connecting PAD and dementia. Moreover, limited mobility and lack of proper self‐care caused by dementia can play a role in the development of PAD (Song et al. 2020; Wardlaw et al. 2019; Jiménez‐Ruiz et al. 2024).

In this analysis, the sex‐specific trends in PAD‐dementia mortality revealed that there was general stability among both sexes regarding the long‐term trend in PAD‐related death, but the more recent male AAMR was marginally higher than that of females. However, females make up a majority of the deaths due to longevity and the tendency of females to live long enough to have both PAD and dementia, because these conditions are highly prevalent at an advanced age. It implies that proportional mortality provides a different perspective of disease incidence in a population compared to age‐adjusted mortality, since sex differences in mortality do not depend only on the severity of diseases among sexes, but population demographics matter. Sex differences in biological processes could play a minor role in mortality, where estrogen depletion after menopause leads to accelerated dysfunction in the endothelium and arterial stiffening (Appelman et al. 2015). But for very old individuals, the contribution from these sex‐specific vascular risk factors becomes less pronounced due to multimorbidity, frailty, and dependency, which become more important determinants of mortality in combined PAD and dementia (Martin et al. 2024). More importantly, the high proportion of deaths among patients institutionalized in LTCFs implies that institutionalization and disabilities play a central role in shaping mortality patterns, as women account for a larger percentage of individuals with dementia and have longer life expectancy despite their disabilities. This evidence implies that the sex differences in PAD‐related dementia mortalities are mainly associated with demographic factors rather than intrinsic biological differences in disease lethality.

Furthermore, it is critical to acknowledge the impact of the COVID‐19 pandemic during the latter years of our study period (2020–2024). The pandemic was associated with disproportionate excess mortality among individuals with dementia, particularly those residing in nursing homes and long‐term care facilities (Hogan et al. 2024; Ghosh et al. 2024). The fluctuations or spikes in AAMRs observed in recent years may partially reflect pandemic‐related vulnerabilities, disruptions in routine vascular care, and changes in death certification practices, rather than solely reflecting long‐term epidemiological trends.

Our study demonstrated that there were significant differences in PAD–dementia deaths across race and ethnicity, with NH African American individuals having the highest AAMR even as it declined over time. The results obtained are aligned with contemporary evidence suggesting that the NH African American population suffers from more vascular complications, increased risk of mortality, and social adversities as compared to the NH White counterparts (Dicks et al. 2023; Allison et al. 2023). Despite the fact that there have been many racial and ethnic groups experiencing reductions in mortality rates throughout the duration of the study, the rise in mortality among NH White individuals and NH Asian and Pacific Islanders indicates that improvements have not been uniform among demographic groups. This may be due to multiple underlying causes related to cardiometabolic risk factors (Allison et al. 2023; McDermott et al. 2023). In addition, the existence of risk factors affecting the underserved population is recognized as an important factor in the pathogenesis of vascular cognitive impairment and dementia caused by cerebrovascular damage, small vessel disease, and atherosclerosis (Chang Wong and Chang Chui 2022; Rundek et al. 2022). Overall, these results suggest that the higher mortality burden observed among certain minority groups could potentially be influenced by disparities in disease prevalence and social determinants of health, though further research is required to establish causality.

Our analysis indicated considerable geographic variability in mortality from PAD‐related dementia in the United States. While Southern states made up the highest proportion of deaths, the Midwest and West had the highest AAMR; conversely, Northeastern states showed consistently low levels of mortality burden that declined during the course of our study.

Furthermore, mortality was higher in non‐metropolitan areas than in metropolitan areas, suggesting urban‐rural differences that are supported well by the current research on geographic disparities in PAD outcomes, access to care, and risk factors in the United States (Allison et al. 2023; McDermott et al. 2023). The increased mortality seen in rural areas may be due to limited access to specialist vascular care, delayed diagnosis, lower utilization of evidence‐based treatments, and higher rates of chronic cardiometabolic conditions (Issa et al. 2023). In addition, differences in the long‐term care system and the available resources, socioeconomic factors, and healthcare utilization may also impact the observed differences in mortality across states and across census regions. All these findings together suggest that geographic variations continue to be an important determinant in the mortality rates associated with PAD–dementia.

The notable finding from our sensitivity analyses included the contrasting trends in mortality for patients with PAD versus dementia. Although mortality related to PAD showed a consistent downward trend over time, dementia‐related mortality steadily rose throughout the observation period. This trend is an indicator of the larger epidemiological shift in chronic diseases experienced by older individuals. Our result concerning improvements in the outcome measures in patients with PAD is consistent with recent literature suggesting marked developments in management through guideline‐based medical therapy and interventions, leading to improved survival (Criqui et al. 2021; Gornik et al. 2024). Intensification of lipid management, antithrombotic approach, and exercise regimens are key aspects of the latest guidelines for PAD by the multi‐society group ACC/AHA in 2024 that have resulted in better vascular health and fewer limb problems. On the other hand, there is still an increasing incidence and death rate of dementia cases worldwide, mainly because of aging populations and the improvement of survival rates of individuals affected by cardiovascular disorders (Li et al. 2022). Moreover, vascular aging and atherogenic burden continue to be among the main pathogenetic pathways through which PAD may affect cognition, in the context of chronic hypoperfusion, endothelial dysfunction, and small vessel cerebrovascular disease (Rajeev et al. 2023). In summary, these studies point to the conclusion that whereas mortality in PAD has benefited from the progress made in cardiology, the growing number of patients suffering from dementia has been a determinant of mortality trends.

## 5 Limitations

There were various limitations to this study. First, the study relied on mortality data from the CDC WONDER database; there were risks of possible misclassification and coding errors that come with using death certificates based on ICD‐based reporting. Second, there was no patient‐specific clinical information included in the database, such as PAD severity, dementia subtype, comorbidities, treatments, and socioeconomic status, thus making the assessment of risk factors and underlying mechanisms impossible. Third, the temporal relationship of PAD to dementia could not be discerned from the mortality data, thus ruling out causal conclusions. Fourth, changes in diagnostic practice, disease awareness, and death certificate reporting over time might have had some effects on the mortality trends. Fifth, our operational definition of PAD utilized a broad set of ICD‐10 codes that included aneurysms, arterial embolism, and unspecified arterial disorders, capturing a wider population than conventional lower‐extremity PAD. Consequently, our findings reflect the mortality burden of a broader category of systemic arterial and peripheral vascular diseases. While this approach effectively captures the systemic nature of atherosclerosis, it may overestimate the mortality specifically attributable to isolated lower‐extremity PAD, and our results must be interpreted within this broader vascular context. Sixth, there is the possibility of erroneous reports when using race and ethnicity data reported on death certificates. Moreover, in the year 1999, there were no reliable estimates for the AAMRs among NH American Indians since they were statistically insignificant; therefore, any information regarding this category of individuals should be interpreted with caution. Further, considering that this study is observational, its findings are only applicable within the United States and other similar demographic and clinical settings. Furthermore, our study exclusively utilizes mortality data, which represents a downstream health outcome. Therefore, our findings are inherently influenced by underlying changes in PAD and dementia incidence and prevalence, as well as improvements in diagnostic practices and survival rates over the 25‐year study period. Without parallel longitudinal incidence data, it is difficult to determine whether the observed mortality trends are driven by changing disease occurrence or evolving case‐fatality rates. Finally, our study design focused on the concurrent burden of both conditions and did not include formal comparator groups, such as age‐matched individuals with isolated PAD, isolated dementia, or the general population. Consequently, while we report the substantial mortality burden associated with their coexistence, we cannot definitively conclude whether this burden is uniquely specific to their synergistic effect or if it primarily reflects the independent mortality contributions of each disease.

## 6 Conclusions

Despite a relatively stable age‐standardized mortality rate, there were clear differences in mortality patterns by demography and geography between 1999 and 2024 for the US adult population with concomitant PAD and dementia. Mortality rates were the highest among males, NH African Americans, and individuals living in non‐metropolitan regions. Importantly, sensitivity analysis showed contrasting mortality patterns, where mortality driven by PAD declined whereas dementia‐related mortality continued to increase. These findings highlight the growing nexus between vascular and cognitive health and the need for equitable access to preventive care and vascular risk factor management, suggesting an integrated vascular and cognitive health strategy.

## Author Contributions

**Naveed Ahmad**: conceptualization, writing – original draft, writing – review and editing, software, data curation, visualization, formal analysis, project administration. **Syed Tawassul Hassan**: conceptualization, writing – original draft, writing – review and editing, supervision, data curation, visualization, formal analysis, project administration. **Hajra Hameed**: writing – original draft, writing – review and editing, validation, visualization, software, methodology, resources. **Muhammad Owais Shaikh**: software, data curation, validation, formal analysis, visualization. **Kaneez Fatima**: data curation, investigation, validation, resources, writing – review and editing. **Aneeza Hameed**: data curation, investigation, validation, resources, writing – review and editing. **Muhammad Affaan Ansari**: project administration, investigation, resources. **Ali Jan Yaqubi**: data curation, project administration, resources.

## Funding

The authors have nothing to report.

## Ethics Statement

This study used publicly available, de‐identified data from the Centers for Disease Control and Prevention Wide‐ranging Online Data for Epidemiologic Research (CDC WONDER) database and did not involve human participants or identifiable private information. As such, institutional ethics approval and informed consent were not required, in accordance with national regulations and institutional policies. The study complies with the ethical standards of the Declaration of Helsinki.

## Conflicts of Interest

The authors declare no conflicts of interest.

## Supporting information




**Supplementary Information**: brb371912‐sup‐0001‐SuppMat.docx

## Data Availability

The datasets generated during and/or analyzed during the current study are publicly available.
