# Supplementary material for: Trends and Disparities in Peripheral Artery Disease and Dementia‐Related Mortality in the United States, 1999–2024
Source: Brain Behav. 2026 Nov 9;16(11):e71912. doi: 10.1002/brb3.71912 (PMC13746988; doi:10.1002/brb3.71912)
Supplement: Supplementary file 1 [file BRB3-16-e71912-s001.docx]

**Supplemental Table 1**; Peripheral artery disease and dementia related mortality, percent total deaths among older adults stratified by overall, gender, race/ ethnicity, place of death, urbanization, and regions in the United States, 1999-2024.

**Supplementary Figure 1.** Percent total deaths of peripheral artery disease and dementia related mortality by place of death among older adults in the United States, 1999 to 2024

**Supplemental Table 2.** Overall and sex-stratified peripheral artery disease and dementia related age-adjusted mortality rates per 100,000 among older adults in the United States, 1999 to 2024.

**Supplemental Table 3.** Annual percent change (APC) of trends in peripheral artery disease and dementia related age-adjusted mortality rates per 100,000 among older adults in the United States, 1999 to 2024.

**Supplemental Table 4.** Race/ Ethnicity stratified trends in peripheral artery disease and dementia related age-adjusted mortality rates per 100,000 among older adults in the United States, 1999 to 2024.

**Supplemental Table 5.** Region-stratified trends in peripheral artery disease and dementia related age-adjusted mortality rates per 100,000 among older adults in the United States, 1999 to 2024

**Supplemental Table 6.** Urbanization stratified trends in peripheral artery disease and dementia related age-adjusted mortality rates per 100,000 among older adults in the United States from 1999 to 2020

**Supplemental Table 7.** State-stratified trends in peripheral artery disease and dementia related age-adjusted mortality rates per 100,000 and their respective percentiles among older adults in the United States, 1999 to 2020.

**Supplemental Table 8.** Sensitivity analysis representing age-adjusted mortality rates per 100,000 population for deaths involving peripheral artery disease and dementia among older adults in the United States, stratified by underlying cause of death and contributing cause of death, 1999-2024.

| **Variables** | **Deaths** | **% of Total Deaths** |
| --- | --- | --- |
| **Overall PAD and dementia** | 317390 | 100.00 |
| **Gender** | | |
| Female | 199012 | 62.70 |
| Male | 118378 | 37.30 |
| **Race/Ethnicity** | | |
| NH Asian | 5282 | 1.66 |
| NH African American | 33210 | 10.46 |
| NH White | 260733 | 82.15 |
| Hispanic | 17086 | 5.38 |
| NH American Indian | 1066 | 0.34 |
| **Place of Deaths** | | |
| Medical Facility | 51228 | 16.14 |
| Decedent's home | 57219 | 18.03 |
| Hospice facility | 11406 | 3.59 |
| Nursing home/long term care | 179518 | 56.56 |
| Other | 17406 | 5.48 |
| Unknown | 608 | 0.19 |
| **Urbanization** | | |
| Metropolitan Areas | 212034 | 79.90 |
| Non-Metropolitan Areas | 53356 | 20.10 |
| **Regions** | | |
| Northeast | 51004 | 16.07 |
| Midwest | 80689 | 25.42 |
| South | 112727 | 35.52 |
| West | 72970 | 22.99 |
| **Sensitivity Analysis** | | |
| PAD as UCD | 675767 | - |
| PAD as UCD + Dementia as Contributing Cause | 63404 | 19.98 |
| Dementia as UCD | 5150367 | - |
| Dementia as UCD + PAD as Contributing Cause | 94885 | 29.90 |

NH: Non-Hispanic; PAD: Peripheral Artery Disease

**Supplemental Table 1**; Peripheral artery disease and dementia related mortality, percent total deaths among older adults stratified by overall, gender, race/ ethnicity, place of death, urbanization, and regions in the United States, 1999-2024.

**Supplementary Figure 1.** Percent total deaths of peripheral artery disease and dementia related mortality by place of death among older adults in the United States, 1999 to 2024

| **Age-Adjusted Rate per 100,000 population (95% Confidence Intervals)** | | | |
| --- | --- | --- | --- |
| **Year** | **Men** | **Women** | **Overall** |
| **1999** | 22.94 (22.05–23.83) | 24.07 (23.43–24.71) | 23.97 (23.45–24.49) |
| **2000** | 38.30 (37.17–39.43) | 39.32 (38.52–40.13) | 39.35 (38.69–40.01) |
| **2001** | 38.21 (37.08–39.33) | 39.39 (38.59–40.20) | 39.28 (38.62–39.93) |
| **2002** | 37.49 (36.39–38.59) | 39.56 (38.75–40.36) | 39.11 (38.46–39.76) |
| **2003** | 37.25 (36.17–38.32) | 38.91 (38.11–39.70) | 38.70 (38.06–39.34) |
| **2004** | 35.90 (34.85–36.95) | 36.46 (35.70–37.22) | 36.57 (35.95–37.19) |
| **2005** | 33.88 (32.88–34.88) | 34.63 (33.89–35.36) | 34.70 (34.11–35.30) |
| **2006** | 30.89 (29.95–31.83) | 30.45 (29.77–31.14) | 30.87 (30.31–31.42) |
| **2007** | 29.68 (28.78–30.59) | 29.39 (28.73–30.06) | 29.72 (29.18–30.26) |
| **2008** | 28.91 (28.03–29.79) | 28.70 (28.04–29.35) | 28.94 (28.41–29.46) |
| **2009** | 27.30 (26.46–28.14) | 26.90 (26.27–27.53) | 27.23 (26.72–27.73) |
| **2010** | 27.44 (26.61–28.28) | 26.32 (25.70–26.94) | 26.95 (26.45–27.44) |
| **2011** | 26.77 (25.96–27.57) | 26.62 (26.00–27.23) | 26.85 (26.36–27.34) |
| **2012** | 26.27 (25.48–27.05) | 25.66 (25.06–26.26) | 26.14 (25.66–26.62) |
| **2013** | 26.03 (25.26–26.81) | 24.89 (24.31–25.47) | 25.50 (25.03–25.96) |
| **2014** | 25.29 (24.54–26.04) | 23.87 (23.30–24.44) | 24.59 (24.14–25.05) |
| **2015** | 24.48 (23.76–25.21) | 22.34 (21.80–22.89) | 23.37 (22.93–23.80) |
| **2016** | 23.40 (22.70–24.10) | 21.63 (21.09–22.16) | 22.50 (22.07–22.92) |
| **2017** | 24.11 (23.41–24.81) | 21.86 (21.33–22.39) | 22.94 (22.52–23.37) |
| **2018** | 23.14 (22.46–23.82) | 21.74 (21.22–22.26) | 22.45 (22.03–22.86) |
| **2019** | 23.69 (23.01–24.36) | 21.64 (21.12–22.16) | 22.57 (22.16–22.99) |
| **2020** | 28.82 (28.09–29.56) | 27.23 (26.66–27.81) | 28.03 (27.58–28.49) |
| **2021** | 27.86 (27.12–28.62) | 26.52 (25.93–27.12) | 27.22 (26.76–27.69) |
| **2022** | 26.37 (25.67–27.10) | 24.30 (23.76–24.85) | 25.27 (24.84–25.71) |
| **2023** | 25.47 (24.78–26.17) | 24.14 (23.59–24.70) | 24.84 (24.41–25.27) |
| **2024** | 24.63 (23.97–25.30) | 23.66 (23.13–24.20) | 24.22 (23.81–24.64) |
| **Mean/Total Average** | **28.64** | **28.08** | **28.53** |

*AAMR = Age-Adjusted Mortality Rate; CI = Confidence Interval.*

**Supplemental Table 2.** Overall and sex-stratified peripheral artery disease and dementia related age-adjusted mortality rates per 100,000 among older adults in the United States, 1999 to 2024.

| **Year Interval** | **APC (95% CI)** | **P-value** | **AAPC (95% CI)** | **P-value** |
| --- | --- | --- | --- | --- |
| **Overall** | | | | |
| 1999-2001 | 21.29 (−0.47 to 47.81) | 0.055154 | −0.23 (−1.86 to 1.43) | 0.785869 |
| 2001-2015 | −4.06* (−4.94 to −3.17) | < 0.000001 |  |  |
| 2015-2024 | 1.54 (−0.11 to 3.23) | 0.066982 |  |  |
| **Gender** | | | | |
| **Men** | | | | |
| 1999-2001 | 23.27* (0.69 to 50.90) | 0.043388 | −0.07 (−1.72 to 1.61) | 0.936627 |
| 2001-2012 | −4.26* (−5.56 to −2.94) | 0.000003 |  |  |
| 2012-2024 | 0.37 (−0.61 to 1.35) | 0.444366 |  |  |
| **Women** | | | | |
| 1999-2001 | 21.99* (0.48 to 48.10) | 0.045145 | −0.34 (−1.96 to 1.30) | 0.683985 |
| 2001-2015 | −4.40* (−5.30 to −3.50) | < 0.000001 |  |  |
| 2015-2024 | 1.66 (−0.09 to 3.44) | 0.061634 |  |  |
| **Race and Ethnicity** | | | | |
| **Hispanic** | | | | |
| 1999–2018 | −1.67 (−2.50 to −0.83) | 0.000563 | −1.23 (−3.82 to 1.43) | 0.361320 |
| 2018–2021 | 7.36 (−12.82 to 32.19) | 0.482918 |  |  |
| 2021–2024 | −6.52 (−15.52 to 3.44) | 0.178527 |  |  |
| **NH American Indian** | | | | |
| 2000-2017 | −3.74* (−4.88 to −2.59) | 0.000003 | −2.51 (−5.99 to 1.09) | 0.169604 |
| 2017-2020 | 15.69 (−13.03 to 53.90) | 0.296209 |  |  |
| 2020-2024 | −9.51* (−16.61 to −1.82) | 0.019322 |  |  |
| **NH Asian or Pacific Islander** | | | | |
| 1999-2001 | 22.08 (−13.88 to 73.05) | 0.241879 | −0.09 (−3.08 to 2.99) | 0.954643 |
| 2001-2017 | −3.01* (−3.97 to −2.05) | 0.000009 |  |  |
| 2017-2022 | 7.63* (2.09 to 13.48) | 0.009655 |  |  |
| 2022-2024 | −13.90 (−26.53 to 0.90) | 0.062661 |  |  |
| **NH African American** | | | | |
| 1999-2002 | 14.31 (−1.38 to 32.49) | 0.073069 | −0.97 (−2.94 to 1.04) | 0.342858 |
| 2002-2015 | −5.07* (−6.55 to −3.56) | 0.000002 |  |  |
| 2015-2024 | 0.36 (−2.14 to 2.92) | 0.770432 |  |  |
| **NH White** | | | | |
| 1999-2001 | 22.07* (2.46 to 45.43) | 0.027849 | −0.12 (−1.58 to 1.36) | 0.874835 |
| 2001-2014 | −4.27* (−5.19 to −3.35) | < 0.000001 |  |  |
| 2014-2024 | 1.40* (0.08 to 2.75) | 0.039232 |  |  |
| **Urbanization** | | | | |
| **Metropolitan Area** | | | | |
| 1999-2001 | 26.22* (15.48 to 37.95) | 0.000126 | 0.04 (−1.09 to 1.18) | 0.942295 |
| 2001-2008 | −5.95* (−7.21 to −4.66) | 0.000001 |  |  |
| 2008-2018 | −2.50* (−3.25 to −1.74) | 0.000019 |  |  |
| 2018-2020 | 11.92* (3.46 to 21.07) | 0.009209 |  |  |
| **Non-metropolitan** | | | | |
| 1999-2001 | 17.62 (−4.86 to 45.43) | 0.124941 | −0.97 (−2.88 to 0.97) | 0.324150 |
| 2001-2020 | −2.75* (−3.32 to −2.18) | < 0.000001 |  |  |
| **Census Regions** | | | | |
| **Northeast Region** | | | | |
| 1999-2001 | 23.74 (−2.35 to 56.81) | 0.075026 | −0.45 (−2.54 to 1.68) | 0.674088 |
| 2001-2007 | −5.30* (−9.68 to −0.712) | 0.026406 |  |  |
| 2007-2024 | −1.24* (−2.01 to −0.48) | 0.003197 |  |  |
| **Midwest region** | | | | |
| 1999-2001 | 29.17* (11.04 to 50.26) | 0.002261 | −0.85 (−2.11 to 0.43) | 0.192134 |
| 2001-2012 | −5.43* (−6.44 to −4.42) | < 0.000001 |  |  |
| 2012-2024 | −0.91* (−1.80 to −0.01) | 0.048278 |  |  |
| **South Region** | | | | |
| 1999-2001 | 17.63 (−2.90 to 42.51) | 0.092188 | −0.50 (−2.09 to 1.12) | 0.545255 |
| 2001-2015 | −4.67* (−5.58 to −3.75) | < 0.000001 |  |  |
| 2015-2024 | 2.47* (0.78 to 4.19) | 0.006403 |  |  |
| **West region** | | | | |
| 1999-2001 | 32.12* (18.77 to 46.97) | 0.000099 | 0.33 (−1.00 to 1.67) | 0.630808 |
| 2001-2007 | −6.60* (−8.43 to −4.73) | 0.000007 |  |  |
| 2007-2018 | −1.17* (−1.86 to −0.48) | 0.003174 |  |  |
| 2018-2021 | 8.98* (0.60 to 18.06) | 0.037314 |  |  |
| 2021-2024 | −6.28* (−9.87 to −2.54) | 0.00357 |  |  |
| **Sensitivity Analysis** | | | | |
| **PAD as UCD** | | | | |
| 1999–2004 | −3.81* (−4.99 to −2.15) | 0.001200 | −4.77* (−5.29 to −4.26) | <0.000001 |
| 2004–2011 | −8.63* (−10.19 to −7.75) | <0.000001 |  |  |
| 2011–2024 | −3.01* (−3.44 to −2.50) | <0.000001 |  |  |
| **PAD as UCD + Dementia as Contributing Cause** | | | | |
| 1999–2004 | 3.54* (0.84 to 8.15) | 0.016397 | −4.61* (−6.55 to −2.64) | 0.000006 |
| 2004–2007 | −18.95* (−21.69 to −12.10) | <0.000001 |  |  |
| 2007–2024 | −4.17* (−4.89 to −3.26) | <0.00000 |  |  |
| **Dementia as UCD** | | | | |
| 1999–2010 | 7.15* (6.36 to 8.22) | <0.000001 | 3.77* (3.37 to 4.18) | <0.000001 |
| 2010–2024 | 1.19* (0.78 to 1.59) | <0.000001 |  |  |
| **Dementia as UCD + PAD as Contributing Cause** | | | | |
| 1999–2007 | 7.89* (5.27 to 13.84) | <0.000001 | 4.06* (2.72 to 5.42) | <0.000001 |
| 2007–2024 | 1.94* (1.08 to 2.58) | 0.005599 |  |  |

*Abbreviations: APC = Annual Percent Change; AAPC = Average Annual Percent Change; CI = Confidence Interval; UCD = Underlying Cause of Death; MCD = Multiple/Contributing Cause of Death.* **Indicates that the Annual Percent Change (APC) is significantly different from zero at* $\alpha=0.05$*.*

**Supplemental Table 3.** Annual percent change (APC) of trends in peripheral artery disease and dementia related age-adjusted mortality rates per 100,000 among older adults in the United States, 1999 to 2024.

| **Age-Adjusted Rate /100,000 (95% CI)** | | | | | |
| --- | --- | --- | --- | --- | --- |
| **Year** | **NH White** | **NH African American** | **NH American Indian** | **Hispanic** | **NH Asian** |
| **1999** | 23.6(23.1-24.2) | 34.1(31.9-36.4) | 32.1(22.5-44.4) | 17.9(15.6-20.2) | 10.7(8.1-13.8) |
| **2000** | 39.2(38.5-39.9) | 52.3(49.5-55) | 30.3(20.8-42.5) | 29.4(26.5-32.4) | 15.7(12.7-19.2) |
| **2001** | 39.3(38.6-40) | 51.4(48.7-54.2) | 26.7(18.2-37.9) | 27.4(24.7-30.2) | 17.6(14.4-20.9) |
| **2002** | 38.8(38.1-39.5) | 55.9(53.1-58.8) | 28.1(19.4-39.2) | 28.5(25.8-31.3) | 15.7(12.8-18.6) |
| **2003** | 38.1(37.5-38.8) | 58.1(55.2-61) | 35.3(25.4-47.7) | 28.9(26.2-31.6) | 17.3(14.3-20.3) |
| **2004** | 36(35.3-36.6) | 56(53.2-58.9) | 24.3(16.3-34.9) | 26.2(23.7-28.7) | 16.9(14-19.7) |
| **2005** | 34.2(33.6-34.9) | 50.8(48.1-53.4) | 28.3(19.7-39.3) | 27.7(25.2-30.1) | 14.8(12.2-17.3) |
| **2006** | 30.4(29.8-31) | 44.4(41.9-46.9) | 27.4(19.1-38.1) | 24.8(22.6-27.1) | 14.7(12.3-17.1) |
| **2007** | 29.3(28.7-29.8) | 45.7(43.2-48.1) | 20.4(13.5-29.4) | 23.2(21-25.3) | 13.5(11.2-15.7) |
| **2008** | 28.4(27.8-28.9) | 41.8(39.5-44.2) | 21.5(14.6-30.5) | 26.8(24.6-29.1) | 13.6(11.4-15.8) |
| **2009** | 26.9(26.3-27.4) | 40.1(37.8-42.3) | 23.9(16.6-33.2) | 22.3(20.4-24.3) | 13.2(11.1-15.3) |
| **2010** | 26.5(26-27) | 38.3(36.1-40.5) | 22.1(15.3-30.9) | 25.3(23.3-27.4) | 13.4(11.3-15.4) |
| **2011** | 26.7(26.2-27.3) | 36.7(34.6-38.8) | 19.6(13.5-27.6) | 24.6(22.7-26.5) | 10.7(8.9-12.4) |
| **2012** | 25.9(25.4-26.5) | 36.1(34-38.1) | 19.8(13.9-27.4) | 23.7(21.9-25.5) | 12.9(11-14.7) |
| **2013** | 25.3(24.8-25.8) | 34.5(32.5-36.5) | 21.3(15.2-28.9) | 24.7(22.9-26.5) | 11.8(10.2-13.5) |
| **2014** | 24.5(24-25) | 32.1(30.3-34) | 21.1(15.2-28.5) | 23.3(21.6-25) | 12.5(10.9-14.2) |
| **2015** | 23.5(23-24) | 30.2(28.5-32) | 14.9(10.2-20.9) | 20.9(19.3-22.4) | 11.3(9.8-12.8) |
| **2016** | 22.6(22.1-23) | 29.5(27.7-31.2) | 15.9(11.2-22.1) | 20.8(19.3-22.4) | 10.9(9.4-12.3) |
| **2017** | 23.2(22.8-23.7) | 29.5(27.8-31.2) | 21.9(16.4-28.7) | 19.8(18.3-21.2) | 10.5(9.1-11.9) |
| **2018** | 22.7(22.2-23.1) | 27.6(26-29.2) | 18.7(13.8-24.8) | 20.3(18.9-21.7) | 12.4(10.9-13.8) |
| **2019** | 23.1(22.6-23.5) | 26.6(25.1-28.2) | 27.6(21.7-34.5) | 19.9(18.6-21.3) | 10.9(9.6-12.2) |
| **2020** | 28(27.5-28.5) | 38(36.2-39.8) | 26(20.1-33.1) | 26.6(25-28.1) | 13.2(11.8-14.6) |
| **2021** | 27.5(26.9-28) | 33.6(31.8-35.4) | 19.9(15-25.9) | 25.5(23.9-27.1) | 14.6(13.1-16.2) |
| **2022** | 25.6(25.1-26.1) | 30.7(29.1-32.4) | 20(15.2-25.8) | 22.9(21.5-24.4) | 15.5(14-17) |
| **2023** | 25.5(25.1-26.1) | 29.3(27.7-31) | 17.9(13.6-23.1) | 21.9(20.6-23.4) | 12.8(11.5-14.2) |
| **2024** | 24.9(24.5-25.4) | 28.8(27.2-30.4) | 32.1(22.5-44.4) | 21.6(20.3-23) | 11.5(10.3-12.8) |
| **Mean/Total Average** | 28.5 | 38.9 | 23.4 | 24.0 | 13.4 |

*NH = Non-Hispanic; AAMR = Age-Adjusted Mortality Rate; CI = Confidence Interval.*

**Supplemental Table 4.** Race/ Ethnicity stratified trends in peripheral artery disease and dementia related age-adjusted mortality rates per 100,000 among older adults in the United States, 1999 to 2024.

| **Age-Adjusted Rate /100,000 (95% CI)** | | | | | | |
| --- | --- | --- | --- | --- | --- | --- |
| **Year** | **Northeast** | **Midwest** | | **South** | **West** | |
| **1999** | 17.78 (16.82–18.74) | | 25.57 (24.5–26.65) | 28.35 (27.39–29.32) | | 21.11 (20–22.23) |
| **2000** | 29.49 (28.27–30.71) | | 45.84 (44.4–47.28) | 40.11 (38.97–41.25) | | 40.98 (39.45–42.51) |
| **2001** | 29.6 (28.38–30.81) | | 46.06 (44.62–47.49) | 39.71 (38.58–40.83) | | 40.93 (39.42–42.44) |
| **2002** | 28.69 (27.5–29.88) | | 45.6 (44.18–47.02) | 40.77 (39.63–41.91) | | 39.81 (38.33–41.28) |
| **2003** | 27.13 (25.99–28.28) | | 46.35 (44.94–47.77) | 39.51 (38.4–40.62) | | 40.7 (39.23–42.17) |
| **2004** | 27.24 (26.1–28.39) | | 42.54 (41.18–43.89) | 38.06 (36.98–39.14) | | 36.79 (35.41–38.18) |
| **2005** | 25.14 (24.06–26.23) | | 40.8 (39.49–42.12) | 36.65 (35.6–37.69) | | 34.17 (32.86–35.49) |
| **2006** | 23.93 (22.88–24.98) | | 36.41 (35.18–37.64) | 30.9 (29.95–31.85) | | 31.24 (30–32.47) |
| **2007** | 22.69 (21.67–23.71) | | 35.43 (34.22–36.63) | 30.26 (29.33–31.19) | | 29.56 (28.37–30.74) |
| **2008** | 22.66 (21.65–23.67) | | 33.92 (32.75–35.08) | 29.16 (28.26–30.06) | | 29.34 (28.18–30.51) |
| **2009** | 20.91 (19.95–21.87) | | 31.02 (29.91–32.14) | 27.94 (27.07–28.82) | | 28.02 (26.9–29.15) |
| **2010** | 22.34 (21.35–23.33) | | 29.4 (28.32–30.48) | 27.44 (26.58–28.3) | | 28.02 (26.9–29.13) |
| **2011** | 22.93 (21.94–23.93) | | 28.84 (27.79–29.89) | 26.65 (25.82–27.48) | | 28.83 (27.73–29.94) |
| **2012** | 22.45 (21.48–23.43) | | 28.64 (27.6–29.69) | 25.45 (24.66–26.25) | | 27.95 (26.88–29.02) |
| **2013** | 20.21 (19.29–21.13) | | 27.21 (26.2–28.22) | 25.32 (24.54–26.11) | | 29.1 (28.01–30.18) |
| **2014** | 20.2 (19.29–21.12) | | 26.88 (25.89–27.88) | 23.53 (22.78–24.27) | | 27.82 (26.78–28.86) |
| **2015** | 18.43 (17.56–19.3) | | 26.38 (25.39–27.36) | 21.84 (21.14–22.55) | | 27.03 (26.02–28.04) |
| **2016** | 18.16 (17.29–19.02) | | 24.71 (23.77–25.66) | 21.28 (20.59–21.97) | | 26.14 (25.16–27.12) |
| **2017** | 18.68 (17.82–19.54) | | 24.18 (23.26–25.11) | 22.17 (21.47–22.86) | | 26.64 (25.66–27.62) |
| **2018** | 17.78 (16.95–18.62) | | 24.3 (23.38–25.22) | 21.53 (20.85–22.2) | | 26.23 (25.27–27.19) |
| **2019** | 18.05 (17.22–18.89) | | 23.33 (22.43–24.23) | 22.05 (21.38–22.73) | | 26.7 (25.74–27.65) |
| **2020** | 23.03 (22.09–23.98) | | 28.51 (27.52–29.5) | 28.21 (27.46–28.97) | | 31.69 (30.66–32.72) |
| **2021** | 20.43 (19.53–21.37) | | 26.51 (25.53–27.52) | 27.07 (26.31–27.85) | | 34.24 (33.13–35.38) |
| **2022** | 18.75 (17.91–19.62) | | 24.3 (23.39–25.23) | 25.82 (25.11–26.55) | | 30.98 (29.97–32.02) |
| **2023** | 18.41 (17.58–19.28) | | 24.2 (23.28–25.14) | 25.56 (24.85–26.29) | | 29.7 (28.7–30.72) |
| **2024** | 17.46 (16.66–18.29) | | 23.53 (22.65–24.45) | 25.76 (25.06–26.48) | | 27.95 (27.01–28.92) |
| **Mean/Total Average** | **21.74** | | **31.51** | **29.04** | | **31.54** |

*AAMR = Age-Adjusted Mortality Rate; CI = Confidence Interval.*

**Supplemental Table 5.** Region-stratified trends in peripheral artery disease and dementia related age-adjusted mortality rates per 100,000 among older adults in the United States, 1999 to 2024

| **Age-Adjusted Rate /100,000 (95% CI)** | | |
| --- | --- | --- |
| **Year** | **Nonmetropolitan** | **Metropolitan** |
| **1999** | 26.42 (25.19–27.65) | 23.4 (22.83–23.98) |
| **2000** | 41.73 (40.2–43.27) | 38.76 (38.02–39.49) |
| **2001** | 39.55 (38.06–41.04) | 39.25 (38.53–39.98) |
| **2002** | 41.74 (40.21–43.27) | 38.49 (37.77–39.2) |
| **2003** | 40.88 (39.37–42.39) | 38.22 (37.51–38.92) |
| **2004** | 39.6 (38.12–41.08) | 35.91 (35.23–36.59) |
| **2005** | 38.66 (37.21–40.12) | 33.76 (33.11–34.41) |
| **2006** | 34.13 (32.77–35.48) | 30.03 (29.42–30.64) |
| **2007** | 35 (33.64–36.36) | 28.54 (27.96–29.12) |
| **2008** | 34.02 (32.69–35.36) | 27.8 (27.23–28.37) |
| **2009** | 32.6 (31.29–33.9) | 26.05 (25.5–26.59) |
| **2010** | 31.02 (29.75–32.28) | 26.03 (25.49–26.57) |
| **2011** | 29.95 (28.72–31.17) | 26.2 (25.67–26.74) |
| **2012** | 31.26 (30.02–32.51) | 25.01 (24.49–25.52) |
| **2013** | 29 (27.81–30.19) | 24.75 (24.24–25.26) |
| **2014** | 27.31 (26.17–28.46) | 24 (23.5–24.49) |
| **2015** | 27.4 (26.26–28.55) | 22.52 (22.04–22.99) |
| **2016** | 25.55 (24.45–26.65) | 21.9 (21.44–22.36) |
| **2017** | 26.1 (25–27.2) | 22.25 (21.79–22.71) |
| **2018** | 24.64 (23.58–25.7) | 21.99 (21.54–22.45) |
| **2019** | 25.07 (24.01–26.13) | 22.03 (21.58–22.47) |
| **2020** | 30.46 (29.3–31.63) | 27.56 (27.06–28.05) |
| **Total** | **32.00 (31.73–32.27)** | **27.73 (27.61–27.85)** |

*AAMR = Age-Adjusted Mortality Rate; CI = Confidence Interval.*

**Supplemental Table 6.** Urbanization stratified trends in peripheral artery disease and dementia related age-adjusted mortality rates per 100,000 among older adults in the United States from 1999 to 2020

| **State** | **Age-Adjusted Rate (95% CI) 2020** | **Percentile %** |
| --- | --- | --- |
| Nevada | 14.31 (13.35–15.28) | 0 |
| Louisiana | 17.08 (16.35–17.82) | 2 |
| Arkansas | 18.47 (17.59–19.35) | 4 |
| Massachusetts | 19.17 (18.60–19.73) | 6 |
| Florida | 19.35 (19.04–19.66) | 8 |
| New York | 19.58 (19.24–19.92) | 10 |
| Hawaii | 20.03 (18.77–21.29) | 12 |
| Wyoming | 20.99 (18.68–23.30) | 14 |
| Arizona | 21.35 (20.69–22.01) | 16 |
| New Jersey | 22.06 (21.52–22.60) | 18 |
| New Mexico | 22.47 (21.24–23.70) | 20 |
| Delaware | 22.75 (20.97–24.53) | 22 |
| Mississippi | 23.04 (21.99–24.08) | 24 |
| Kansas | 23.10 (22.13–24.07) | 26 |
| Virginia | 23.18 (22.53–23.83) | 28 |
| Connecticut | 23.24 (22.41–24.08) | 30 |
| Illinois | 23.81 (23.32–24.30) | 32 |
| Georgia | 24.21 (23.54–24.88) | 34 |
| Maine | 24.33 (22.94–25.72) | 36 |
| Pennsylvania | 24.56 (24.12–25.00) | 38 |
| Montana | 24.87 (23.17–26.57) | 40 |
| Missouri | 25.62 (24.91–26.33) | 42 |
| Idaho | 25.71 (24.19–27.24) | 44 |
| Alabama | 26.18 (25.33–27.03) | 46 |
| South Dakota | 27.84 (25.96–29.72) | 48 |
| Wisconsin | 28.74 (27.98–29.51) | 50 |
| North Dakota | 29.25 (27.17–31.32) | 52 |
| Alaska | 29.81 (26.28–33.35) | 54 |
| Utah | 29.84 (28.38–31.31) | 56 |
| Kentucky | 30.05 (29.07–31.02) | 58 |
| Iowa | 30.12 (29.13–31.12) | 60 |
| North Carolina | 30.27 (29.60–30.94) | 62 |
| Michigan | 31.52 (30.89–32.14) | 64 |
| Nebraska | 31.75 (30.35–33.15) | 66 |
| Colorado | 31.76 (30.75–32.77) | 68 |
| New Hampshire | 32.02 (30.30–33.75) | 70 |
| District of Columbia | 32.72 (29.98–35.46) | 72 |
| South Carolina | 32.84 (31.85–33.84) | 74 |
| Oregon | 32.93 (31.92–33.95) | 76 |
| Indiana | 33.08 (32.27–33.90) | 78 |
| California | 33.20 (32.84–33.55) | 80 |
| Minnesota | 34.37 (33.49–35.25) | 82 |
| Washington | 34.50 (33.67–35.34) | 84 |
| Rhode Island | 36.38 (34.51–38.25) | 86 |
| Maryland | 37.22 (36.28–38.16) | 88 |
| West Virginia | 37.53 (36.02–39.03) | 90 |
| Texas | 37.75 (37.24–38.26) | 92 |
| Vermont | 40.86 (38.13–43.60) | 94 |
| Tennessee | 41.97 (41.02–42.92) | 96 |
| Oklahoma | 43.04 (41.82–44.27) | 98 |
| Ohio | 48.50 (47.80–49.20) | 100 |

*AAMR = Age-Adjusted Mortality Rate; CI = Confidence Interval.*

**Supplemental Table 7.** State-stratified trends in peripheral artery disease and dementia related age-adjusted mortality rates per 100,000 and their respective percentiles among older adults in the United States, 1999 to 2020.

.

| **Age-Adjusted Rate /100,000 (95% CI)** | | | | |
| --- | --- | --- | --- | --- |
| **Sensitivity Analysis** | | | | |
| **Year** | **PAD as UCD** | **PAD as UCD + Dementia as Contributing Cause** | **Dementia as UCD** | **Dementia as UCD + PAD as Contributing Cause** |
| **1999** | 114.54 (113.41–115.67) | 7.90 (7.60–8.19) | 214.21 (212.66–215.76) | 4.26 (4.04–4.48) |
| **2000** | 111.76 (110.65–112.87) | 10.86 (10.51–11.21) | 237.30 (235.68–238.92) | 5.00 (4.76–5.23) |
| **2001** | 108.02 (106.93–109.10) | 10.75 (10.41–11.09) | 258.16 (256.48–259.83) | 5.01 (4.78–5.25) |
| **2002** | 104.68 (103.61–105.74) | 10.55 (10.22–10.89) | 281.73 (279.99–283.47) | 5.26 (5.02–5.50) |
| **2003** | 100.03 (99.00–101.06) | 10.71 (10.37–11.05) | 299.87 (298.09–301.65) | 5.64 (5.39–5.88) |
| **2004** | 93.67 (92.68–94.66) | 10.23 (9.91–10.56) | 305.60 (303.81–307.38) | 5.39 (5.15–5.62) |
| **2005** | 91.16 (90.19–92.13) | 9.92 (9.60–10.24) | 338.76 (336.90–340.62) | 5.78 (5.54–6.02) |
| **2006** | 76.39 (75.51–77.27) | 6.65 (6.39–6.91) | 385.85 (383.89–387.81) | 8.25 (7.97–8.54) |
| **2007** | 71.27 (70.43–72.11) | 6.15 (5.91–6.40) | 388.40 (386.45–390.34) | 7.85 (7.57–8.12) |
| **2008** | 65.28 (64.48–66.08) | 5.80 (5.57–6.04) | 435.94 (433.90–437.98) | 8.51 (8.23–8.80) |
| **2009** | 60.27 (59.51–61.03) | 5.47 (5.24–5.69) | 423.07 (421.08–425.06) | 8.24 (7.96–8.52) |
| **2010** | 58.31 (57.57–59.05) | 5.45 (5.23–5.68) | 459.69 (457.63–461.74) | 8.43 (8.15–8.71) |
| **2011** | 50.95 (50.27–51.64) | 4.84 (4.63–5.05) | 482.43 (480.35–484.51) | 8.80 (8.52–9.08) |
| **2012** | 49.31 (48.64–49.97) | 4.64 (4.43–4.84) | 494.07 (491.99–496.15) | 8.76 (8.48–9.03) |
| **2013** | 48.54 (47.89–49.19) | 4.76 (4.56–4.96) | 505.32 (503.25–507.40) | 8.95 (8.67–9.22) |
| **2014** | 46.79 (46.16–47.42) | 4.44 (4.25–4.63) | 506.83 (504.77–508.89) | 8.93 (8.65–9.20) |
| **2015** | 45.68 (45.06–46.29) | 4.17 (3.99–4.36) | 508.49 (506.45–510.54) | 8.33 (8.07–8.59) |
| **2016** | 43.25 (42.66–43.85) | 3.98 (3.80–4.15) | 506.53 (504.51–508.55) | 8.18 (7.92–8.43) |
| **2017** | 41.88 (41.30–42.46) | 3.84 (3.67–4.01) | 520.95 (518.93–522.98) | 8.50 (8.24–8.76) |
| **2018** | 39.60 (39.05–40.15) | 3.59 (3.42–3.75) | 519.32 (517.32–521.32) | 8.74 (8.49–9.00) |
| **2019** | 37.35 (36.82–37.88) | 3.24 (3.09–3.40) | 519.39 (517.41–521.37) | 9.24 (8.98–9.51) |
| **2020** | 36.98 (36.46–37.50) | 3.54 (3.38–3.71) | 572.05 (569.99–574.10) | 10.94 (10.65–11.22) |
| **2021** | 38.56 (38.02–39.11) | 3.43 (3.27–3.60) | 568.02 (565.89–570.16) | 11.21 (10.91–11.52) |
| **2022** | 36.88 (36.36–37.40) | 3.20 (3.05–3.36) | 551.75 (549.73–553.77) | 10.34 (10.06–10.62) |
| **2023** | 36.34 (35.83–36.85) | 3.17 (3.01–3.33) | 547.69 (545.66–549.72) | 10.69 (10.40–10.97) |
| **2024** | 34.63 (34.15–35.13) | 2.86 (2.72–3.01) | 548.87 (546.89–550.87) | 10.96 (10.68–11.24) |
| **Mean/Total Average** | **63.16** | **5.93** | **437.70** | **8.08** |

*AAMR = Age-Adjusted Mortality Rate; CI = Confidence Interval; UCD = Underlying Cause of Death; MCD = Multiple/Contributing Cause of Death.*

**Supplemental Table 8.** Sensitivity analysis representing age-adjusted mortality rates per 100,000 population for deaths involving peripheral artery disease and dementia among older adults in the United States, stratified by underlying cause of death and contributing cause of death, 1999-2024.
